# Longitudinal assessment of cerebral infarcts and small vessel disease using magnetic resonance imaging in antiphospholipid syndrome: A single‐centre retrospective study

**DOI:** 10.1002/jha2.1065

**Published:** 2025-02-06

**Authors:** Yishi Tan, Andrew J. Doyle, Jayant Kumar, Peter Somerville, Uzma Faruqi, Anicee Danaee, Pu‐Lin Luo, Beverley J. Hunt, Karen A. Breen

**Affiliations:** ^1^ Haemostasis and Thrombosis Centre St Thomas’ Hospital London UK; ^2^ Department of Radiology St Thomas’ Hospital London UK; ^3^ Department of Stroke Medicine St Thomas’ Hospital London UK

**Keywords:** anticoagulation, antiphospholipid syndrome, imaging, MRI, stroke

## Abstract

**Introduction:**

Stroke is the most frequent arterial thrombosis in antiphospholipid syndrome (APS) with high rates of recurrence.

**Methods and patients:**

A retrospective, single‐centre 10‐year review of patients with APS having sequential cerebral magnetic resonance imaging (MRI) was performed to describe ischaemic features in APS and associated disease risk factors and progression over time.

**Results:**

A total of 120 patients and 307 scans were included with 67% of patients receiving vitamin K antagonists (VKA). Note that 65% of patients had baseline ischaemic features with white matter hyperintensities (WMH), as a feature of small vessel disease (SVD), seen in 79% of abnormal scans. Fifteen percent of patients had progressive ischaemic changes with 83% demonstrating progressive WMH and 33% new infarcts (predominantly lacunar) on sequential scans. Progression‐free survival for progressive ischaemia was 88% at 5 years. Multivariate analysis showed longer follow‐up was a risk for developing progressive ischaemia (odds ratio [OR] 1.43, 95% confidence interval [CI] 1.13–1.86, *p* = 0.005). Hypertension (56% vs. 30%, *p* = 0.04) and ischaemic heart disease (22% vs. 6%, *p* = 0.04) were more prevalent with progressive ischaemia. There was no difference in progression or bleeding events according to VKA therapeutic intensity.

**Discussion:**

These results show SVD is a common feature of APS using MRI with progressive changes despite anticoagulation. Traditional risk factors for cerebrovascular disease were associated with progression.

## 1 BACKGROUND

Antiphospholipid syndrome (APS) is an autoimmune condition characterised by thrombotic and obstetric manifestations, together with the presence of persistent antiphospholipid antibodies (aPL) with anti‐β2‐glycprotein‐1 antibodies being most significant in thrombosis. Stroke is recognised as the most common site of arterial thromboembolism (ATE) in APS [1, 2]. High rates of thrombosis recurrence are seen in APS, particularly with ATE [3]. There is a recognised tendency for thrombosis recurrence at the same vascular site [4].

Neurological manifestations of APS can be diverse [5]. APS is reported to account for 15% of stroke in patients <55 years [6] and the presence of persistent aPL in young adults confers a fivefold increased risk of stroke or TIAs [7, 8]. Neurological presentations such as migraine, seizures and cognitive impairment are recognised in APS [9], which are also recognised in patients with cerebral small vessel disease (SVD) [10]. The pathogenesis of this is felt to be thrombotic [11], although other non‐thrombotic mechanisms have also been postulated [12].

The most common neurological features shown on magnetic resonance imaging (MRI) in APS are consistent with small and large vessel infarcts, with an increasing recognition that white matter hyperintensities (WMH), also known as leukoaraiosis, are relevant [13, 14, 15, 16, 17]. Current management in APS with stroke or cerebral infarcts is the use of vitamin K anticoagulants (VKA). Further ischaemic changes can occur despite anticoagulation in APS and optimal anticoagulation targets are yet to be defined.

The aims of this study were to describe the changes on cerebral MRI in a cohort of patients with APS and assess for progressive ischaemia‐like changes over time with associated risk factors. We hypothesise that new or progressive changes occur despite long‐term antithrombotic treatment in APS.

## 2 METHODS

### 2.1 Study design

This was a single‐centre, retrospective cohort study. Clinical details were obtained from electronic patient records software (iSOFT Group plc) for patients identified with having persistent aPL at the study site. Images were included for review if performed between 1 September 2012 and 31 August 2022.

Inclusion criteria were age ≥18 years, fulfilment of the clinical and laboratory criteria of the revised Sapporo classification of APS [1] and two or more cerebral MRIs performed. Patients were excluded if aPL were not persistent or if less than two cerebral MRI scans were performed between the study dates.

Details of APS‐defining clinical events of thrombosis and/or obstetric morbidity were obtained from electronic patient records and radiological reports (picture archiving and communication system (PACS), Sectra and Sweden). Patients were reviewed a minimum of once annually as part of routine clinical care including laboratory testing. MRI scans were either performed because of (1) the presence of neuro‐cognitive symptoms or (2) as surveillance for previously known cerebrovascular changes.

### 2.2 Outcome measures

The primary outcome was the presence of new or progressive changes on MRI imaging consistent with cerebral ischaemia. Baseline MRI was defined as the first MRI image performed for each patient during the study period with progressive changes defined as changes occurring between the baseline and any subsequent scan in the study period. Progressive ischaemia was defined as either MRI changes consistent with (1) new or an increase in number or features of SVD or (2) new large vessel infarcts. SVD was reviewed in accordance with Standards for Reporting Vascular Changes on Neuroimaging (STRIVE 2) recommendations [18]. MRI changes were characterised by their size, location, shape and distribution. Progression‐free survival for new/progressive cerebral ischaemia was assessed. The follow‐up period for each period was considered from the initial MRI scan to last scan.

Secondary outcomes were disease differences in those with cerebral ischaemia at baseline and with progression on sequential MRI compared to those who did not. Features assessed were antithrombotic treatments, presence of cerebrovascular risk factors and aPL patterns.

In the setting of APS with cerebral ischaemia, the efficacy of VKA anticoagulation targets were reviewed as a factor for cerebral ischaemia. Safety outcomes of this were also assessed. Anticoagulation was either (1) standard‐intensity (SIA), defined as a target INR of 2–3 or 2.5–3.5 or (2) high‐intensity anticoagulation (HIA), defined as a target INR of 3–4. Patients were assessed for bleeding in accordance with the recommendations from the International Society of Thrombosis and Haemostasis for clinically relevant non‐major bleeding (CRNMB) and major bleeding [19].

### 2.3 Laboratory methods

aPL were considered in accordance with the revised Sapporo criteria [1]. For this study, chemiluminescent techniques were used for IgG and IgM ACL and AB2P1 with the single highest value measured being used (Machine: BIO‐FLASH; Kit: QUANTA Flash). LA was performed using diluted Russell viper venom time and dilute activated partial thromboplastin time (dAPTT) for non‐anticoagulated patients (Machine: Sysmex CS2000i; Kit: STA‐Staclot dRVV screen and confirm, Diagnostica Stago for drift; PTT‐LA, Diagnostica Stago with addition of Bio/Data Lupus Anticoauglant Confirmation Reagent, Bio/Data Corp for confirmation of dAPTT). Taipan Snake Venom Time/Ecarin Clotting Time ratios were used to assess for presence of LA in patients on VKA [20, 21].

### 2.4 Details of magnetic resonance imaging

MRI examinations were acquired with a 1.5T MRI (Aera/Avanto, Siemens Healthcare) and/or a 3T MRI (Magentom Skyra, Siemens Healthcare). Sequences acquired included diffusion weighted imaging and apparent weighted coefficient, T2‐weighted axial, 2D or 3D fluid attenuated recovery sequence (FLAIR), T1‐weighted imaging (axial or sagittal planes), and a blood‐sensitive sequence. Further details in MRI changes over time are provided in Supporting Information.

MRI reports from PACS were reviewed independently by two clinicians to identify those with interval ischaemia and were subsequently confirmed by a third clinician. Thereafter, the first and subsequent cerebral MRI imaging with interval changes of those with progressive ischaemia were reviewed by an independent neuroradiologist with >10 years’ experience to confirm the presence of progressive ischaemia and their further characterisation. This review was unblinded.

### 2.5 Statistical analysis

Baseline parameters including patient demographics, blood parameters and clinical phenotype were grouped according to disease status. Due to non‐parametric distribution, the Mann–Whitney *U* test was used. Categorical variables were compared using chi square rest or Fisher's exact test depending on sample size. Multiple logistic regression was performed to assess significant variables identified by univariate analysis. Progression‐free survival was assessed using Kaplan–Meier analysis. Statistical significance was considered with *p*‐value < 0.05. Statistical analysis was performed using GraphPad Prism version 9.5.0 for MacOS (GraphPad Software).

## 3 RESULTS

### 3.1 Patient selection

**FIGURE 1:**
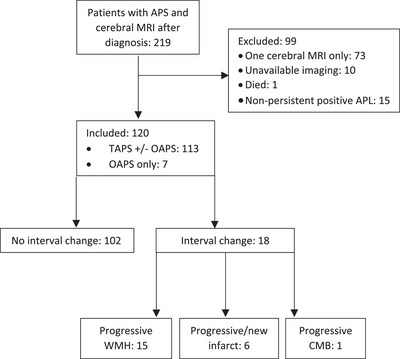
Flow chart of patient inclusion and magnetic resonance imaging (MRI) outcomes. APS, antiphospholipid syndrome; TAPS, thrombotic APS; OAPS, obstetric APS; WMH, white matter hyperintensities; CMB, cerebral microbleeds.

In total, 219 patients were reviewed and 120 were included in the study (Figure 1). Note that 99 were excluded: 73 patients had only one cerebral MRI, 10 patients had external MRIs without available imaging, one died during the study period with no clinical information available and 15 patients did not have documented persistent aPL over the study period. Subsequently, 120 patients with 307 MRI scans were included over a total of 463 patient years (0.7 scan/patient year).

### 3.2 Patient characteristics

The median age was 49.9 years (interquartile range [IQR] 42.0–60.2) at baseline MRI scan. There was a predominance of Caucasian ethnicity (69%) and female gender (83%). Note that 28% of patients had persistent ‘triple positive’ testing. The most common clinical presentation of APS was an ATE preceding the baseline MRI included in the study period (73% overall) (Table 1). The median Adjusted Global Anti‐Phospholipid Syndrome Score (aGAPSS) was 9 (IQR 7–13). Note that 67% of patients received VKA, with 52% of the total cohort having an HIA target.

**TABLE 1:** Comparison of clinical features of patients with cerebral ischaemic features on baseline scan with antiphospholipid syndrome.

	Cerebral ischaemia (*n* = 78)	No ischaemia (*n* = 42)	*p*‐value
**Age** (baseline—years); median (IQR)	52.3 (46.7–61.1)	43.0 (37.0–38.7)	0.005
**BMI** (kg/m^2^); median (IQR)	30.4 (25.8–38.2)	31.1 (27.1–33.2)	0.94
**Ethnicity**			
Caucasian	56/78 (72%)	27/42 (64%)	0.58
Non‐Caucasian	11/78 (11%)	9/42 (21%)	
Not stated	11/78 (14%)	6/42 (14%)	
**Initial clinical presentation**			
Arterial thrombosis	68/78 (87%)	18/42 (43%)	<0.005
Venous thromboembolism	33/78 (42%)	21/42 (50%)	
Obstetric morbidity	9/78 (12%)	14/42 (33%)	
**aPL testing**			
Lupus anticoagulant positive	69/78	34/42	0.28
IgG/IgM ACL positive	42/78	28/42	0.37
Weak positive (10–40 GPL/MPL)	22/78	16/42	0.50
Intermed/high (>40 GPL/MPL)	20/78	12/42	0.98
IgG/IgM Aβ2GP1 positive	31/76	13/41	0.51
Weak positive (10–40 GPL/MPL)	13/76	4/41	0.29
Intermed/high (>40 GPL/MPL)	18/76	9/41	0.47
‘Single positive’	35/76	19/41	0.76
‘Double positive’	18/76	12/41	0.67
‘Triple positive’	23/76	10/41	0.90
IgG ACL titre; median (IQR)	18 (8–98)	30.0 (12–106)	
IgM ACL titre; median (IQR)	18 (10–32)	30.0 (12–96)	
IgG Aβ2GP1 titre; median (IQR)	32 (9–1421)	30 (3–1541)	
IgM Aβ2GP1 titre; median (IQR)	5 (1–19)	3 (2–31)	
**aGAPSS**; median (IQR)	9 (7–13)	9 (5–13)	
**Treatment prior to baseline scan**			
None	10/78 (13%)	5/42 (12%)	0.33
VKA only	52/78 (67%)	25/42 (59.5%)	
Standard‐intensity VKA	11/78 (14%)	6/42 (14%)	
High‐intensity VKA	44/78 (56%)	19/42 (45%)	
DOAC only	2/78 (2.5%)	2/42 (5%)	
LMWH/fondaparinux	4/78 (5%)	2/42 (5%)	
Antiplatelet only	7/78 (9%)	5/42 (12%)	
VKA + antiplatelet	3/78 (4%)	0	
DOAC + antiplatelet	0	0	
LMWH + antiplatelet	0	3/42 (7%)	
**Co‐morbidities**			
Diabetes mellitus	9/78 (11.5%)	2/42 (5%)	0.32
Hyperlipidaemia	10/78 (13%)	2/42 (5%)	0.21
Hypertension	29/78 (37%)	11/42 (3%)	0.31
Ischaemic heart disease	7/78 (9%)	3/42 (7%)	>0.99
Atrial Fibrillation	4/78 (5%)	1/42 (2%)	0.65
SLE	9/78 (11.5%)	10/42 (24%)	0.11
Smoker or ex‐smoker	10/78 (13%)	4/42 (10%)	0.94
**Adjuvant medications**			
Hydroxychloroquine	34/78 (43.5%)	17/42 (40%)	0.74
Statins	37/78 (47%)	9/42 (21%)	0.006

Abbreviations: Aβ2GPI, anti‐β2‐glycoprotein‐I antibody; ACL, anticardiolipin antibody; aGAPSS, Adjusted Global Antiphospholipid Syndrome Score; aPL, antiphospholipid antibody; BMI, body mass index; CRNMB, clinically relevant non major bleeding; DOAC, direct oral anticoagulant; IQR, interquartile range; LMWH, low molecular weight heparin; SLE, systemic lupus erythematosus; SD, standard deviation; VKA, vitamin K antagonist.

### 3.3 Assessment of baseline imaging and features related to ischaemic changes

Note that 78 of 120 (65%) patients have abnormal ischaemic changes on baseline MRI scan. The features of these changes were of SVD in 76 of 78 (94%) patients and large vessel infarcts in six of 78 (8%) patients. Of those with features of SVD, 62 of 76 (82%–79% of all abnormal scans) had WMH, 27 of 76 (36%) had lacunar infarct, six of 76 (8%) had cortical microhaemorrhages, four of 76 (5%) had brain atrophy and two of 76 (3%) had subcortical infarcts. Five of six (86%) patients with large vessel infarcts demonstrated concurrent SVD.

Clinical details of the clinical features with and without baseline ischaemia are shown in Table 1. Age (52.3 vs. 43.0 years, *p* = 0.005) and preceding history of ATE being higher in those with ischaemia (87% vs. 43%, *p* < 0.005). No difference was found in APL testing, use of anticoagulation or other cerebrovascular risk factors.

### 3.4 Assessment of sequential imaging

**FIGURE 2:**
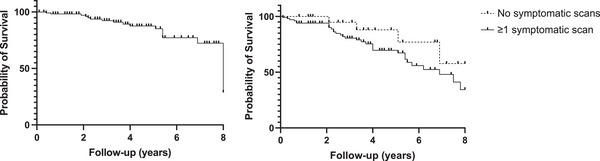
Kaplan–Meier curve of progression‐free survival to development of cerebral ischaemia: (A) all patients; (B) patients with asymptomatic scans.

Note that 102 of 120 (85%) patients had no further changes during follow‐up. The median duration between baseline and last scan was 3.6 years (IQR 2.0–5.5), with an annual event rate for progression of 3.9 per 100‐patient years. However, 18 of 120 (15%) patients with sequential scans had features of new or progressive ischaemic changes. Note that 15 of 18 patients with new or progressive ischaemia (83%) had preceding changes suggestive of ischaemia on their baseline scan. Figure 2 shows the progression‐free survival for the development of subsequent MRI changes. Changes consistent with cerebral ischaemia were seen in 1.7% of patients at 1 year, 7.6% at 3 years or 12.4% at 5 years of follow‐up shown in Figure 2.

**FIGURE 3:**
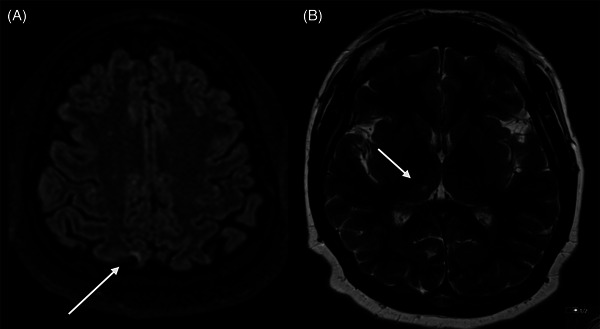
Cerebral magnetic resonance imaging of patients with antiphospholipid syndrome showing infarction: (A) 3D fluid attenuated recovery sequence (FLAIR) image—right parietal gliosis consistent with cortical infarction (arrow); (B) axial T2 image—right thalamic infarct (arrow).

**FIGURE 4:**
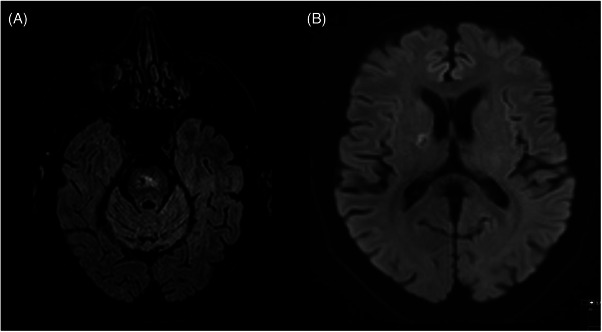
Cerebral magnetic resonance imaging of patients with antiphospholipid syndrome showing infarction: (A) axial T2 image—central pontine gliosis consistent with infarction and (B) axial diffusion weighted imaging (DWI) image with signal abnormality right internal capsule consistent with capsular infarction.

**FIGURE 5:**
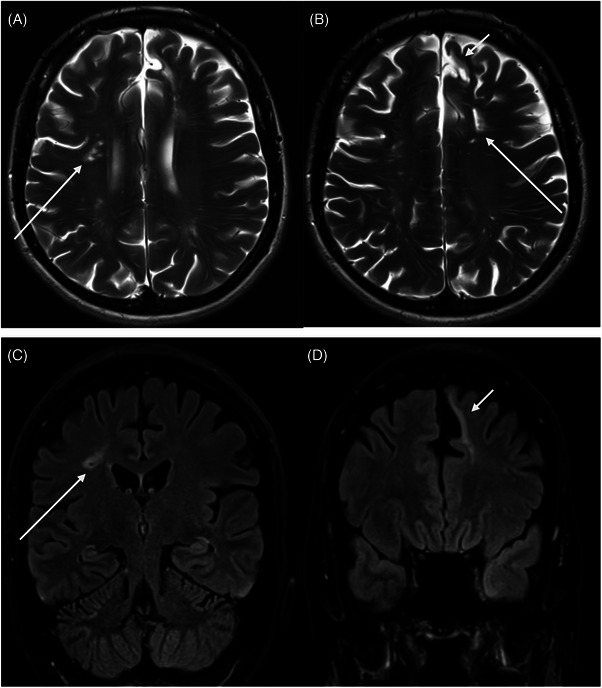
Cerebral magnetic resonance imaging of patients with antiphospholipid syndrome showing infarction. Deep watershed and separate cortical embolic infarcts by (A and B) T2 axial and (C and D) 2D fluid attenuated recovery sequence (FLAIR). Long arrows demonstrate deep watershed hyperintense foci (A and B) which were subsequently supressed on FLAIR imaging, confirming gliotic change secondary to infarction (C). Short arrows outline cortical infarction of the left anterior cerebral territory within the superior frontal gyrus (B and D).

**FIGURE 6:**
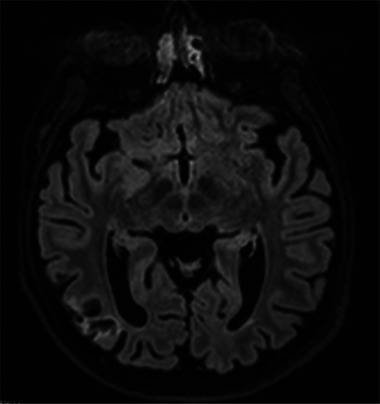
Cerebral magnetic resonance imaging of patients with antiphospholipid syndrome showing watershed infarction—3D fluid attenuated recovery sequence (FLAIR) sequence confirming right external watershed territory infarct interposed between middle and posterior cerebral arterial territories.

**FIGURE 7:**
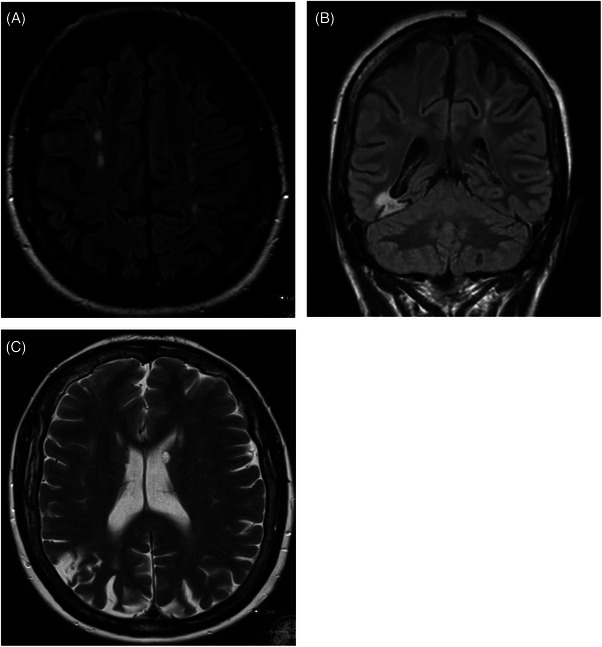
Serial cerebral magnetic resonance imaging of a patient with antiphospholipid syndrome over a 10‐year period. (A) Initial axial fluid attenuated recovery sequence (FLAIR) sequence demonstrating deep watershed infarcts (B) coronal FLAIR sequence acquired 5 years after the initial scan, demonstrating a right occipitotemporal (posterior cerebral artery) territory embolic infarct. (C) Axial T2 sequence acquired 10‐years after the initial scan, demonstrating an external watershed infarct interposed between the middle and posterior cerebral arterial territories.

The radiological details of progressive ischaemic changes seen are described in Table 2. The most frequent MRI changes described were an excess of WMH for age on 14 of 18 (78%) on baseline scans. Similarly, further WMH were seen on 15 of 18 (83%) of abnormal sequential scans. Development of infarcts was less frequent, occurring in six of 18 (33%) patients, which were lacunar in nature. Concurrent progressive WMH were present in 50% (3/6) of patient with new infarcts. The infarct distribution was variable between patients and seen in various brain territories. There was a high proportion of infarcts in watershed territories (4/6; 67%). Examples of imaging features are shown in Figures 3, 4, 5, 6, 7.

**TABLE 2:** Features of patients with progressive cerebral ischaemia with antiphospholipid syndrome.

**Baseline ischaemic features**	
None	4/18 (22%)
White matter hyperintensities (WMH)	
Minimal	14/18 (78%)
Mild	5/18 (28%)
Moderate/severe	8/18 (44%)
Linear/wedge shaped infarcts	1/18 (6%)
Site of baseline lacunar infarct	7/18 (39%)
Cortical	4/18 (22%)
Basal ganglia/thalamic	2/18 (11%)
Cerebellar	6/18 (33%)
Presence of cortical microbleeds	2/18 (11%)
**Progressive ischaemic feature**	
New/progressive WMH	15/18 (83%)
Minimal change	11/18 (61%)
Mild change	3/18 (17%)
Moderate/severe change	1/18 (6%)
New lacunar infarcts	6/18 (33%)
Sites of new progressive/lacunar infarcts	
Cortical	3/6 (50%)
Basal ganglia/thalamic	3/6 (50%)
Cerebellar	2/6 (33%)
Features of new/progressive infarcts	
Linear/wedge‐shaped	1/6 (17%)
Watershed	4/6 (67%)
Both progressive WMH and lacunar infarcts	3/18 (17%)
Cortical microbleeds	1/18 (6%)

Abbreviations: MRI, magnetic resonance imaging; IQR, interquartile range; WMH, white matter hyperdensities; CMB, cerebral microhaemorrhages; IQR, interquartile range; aGAPSS, Adjusted Global Antiphospholipid Syndrome Score; IHD, ischaemic heart disease.

### 3.5 Clinical features associated with progressive ischaemic changes

The characteristics of patients with and without progressive ischaemia were compared (Table 3). There was a longer duration of follow‐up of 5.3 years (IQR 3.3–7.3) for patients with interval ischaemia compared to 3.3 years (IQR 1.5–5.3) for those without (*p* = 0.01). There was no statistically significant difference in the presenting clinical features of APS (ATE vs. VTE vs. obstetric morbidity), aPL testing, aGAPSS and antithrombotic therapy. Six patients with new cerebral lacunar infarcts had a high prevalence of these described risk factors: median aGAPSS 13 (IQR 8–14), triple‐positive aPL 4/6 (67%) and hypertension 4/6 (67%).

**TABLE 3:** Comparison of characteristics between patients with and without progressive ischaemia.

	Progressive ischaemia (*n* = 18)	No progressive ischaemia (*n* = 102)	*p*‐value
**Age** (baseline—years); median (IQR)	50.4 (42.0–60.9)	49.8 (42.0–60.0)	0.91
**Baseline to last scan** (years); median (IQR)	5.3 (3.3–7.3)	3.3 (1.5–5.3)	0.01
**Presence of baseline cerebral ischaemia**	14/18 (78%)	64/102 (63%)	0.29
**BMI** (kg/m^2^); median (IQR)	34.0 (29.7–36.8)	30.4 (26.6–34.4)	0.28
**Ethnicity**			
Caucasian	14/18 (78%)	69/102 (68%)	0.16
Non‐Caucasian	4/18 (22%)	16/102 (16%)	
Not stated	0	17/102 (17%)	
**Preceding clinical presentation**			
Arterial thrombosis	14/18 (78%)	72/102 (71%)	0.75
Venous thrombosis	8/18 (44%)	46/102 (45%)	
Obstetric morbidity	5/18 (28%)	18/102 (18%)	
**aPL testing**			
Lupus anticoagulant positive	16/18 (89%)	87/102 (85%)	>0.99
IgG/IgM anticardiolipin positive	10/18 (56%)	60/102 (59%)	0.26
Weak positive (10–40 GPL/MPL)	3/18 (17%)	35/102 (34%)	0.32
Intermed/high (>40 GPL/MPL)	7/18 (39%)	25/102 (25%)	0.96
IgG/IgM Aβ2GP1 positive	10/18 (56%)	34/99 (34%)	0.15
Weak positive (10–40 GPL/MPL)	7/18 (39%)	20/99 (20%)	0.30
Intermed/high (>40 GPL/MPL)	3/18 (17%)	14/99 (14%)	0.08
‘Single positive’	8/18 (44%)	46/99 (46.5%)	0.32
‘Double positive’	2/18 (11%)	28/99 (28%)	0.16
‘Triple positive’	8/18 (44%)	25/99 (25%)	0.90
IgG ACL titre; median (IQR)	137 (12–502)	20 (9.1–49)	
IgM ACL titre; median (IQR)	13 (8–17)	18 (9.9–37)	
IgG Aβ2GP1 titre; median (IQR)	193 (19–2799)	23 (4–1492)	
IgM Aβ2GP1 titre; median (IQR)	4 (3–7)	5 (2–23)	
**aGAPSS;** median (IQR)	11 (8‐14)	9 (5–13)	0.14
**Co‐morbidities**			
Diabetes mellitus	3/18 (17%)	8/102 (8%)	0.37
Hyperlipidaemia	1/18 (6%)	10/102 (10%)	>0.99
Hypertension	10/18 (56%)	31/102 (30%)	0.04
Ischaemic heart disease	4/18 (22%)	6/102 (6%)	0.04
Atrial fibrillation	0	5/102 (5%)	>0.99
SLE	4/18 (22%)	15/102 (15%)	0.48
Smoker or ex‐smoker	3/18 (17%)	11/102 (11%)	0.27
**Initial antithrombotic therapy**			
None	1/18 (6%)	14/102 (14%)	0.62
VKA only	13/18 (72%)	64/102 (63%)	0.46
Standard‐intensity VKA	3/13 (23%)	9/64 (14%)	0.44
High‐intensity VKA	10/13 (77%)	55/64 (86%)	0.42
DOAC only	1/18 (6%)	3/102 (3%)	0.48
LMWH/fondaparinux	0	6/102 (6%)	0.59
Antiplatelet only	3/18 (17%)	9/102 (9%)	0.39
VKA + antiplatelet	0	3/102 (3%)	>0.99
DOAC + antiplatelet	0	0	>0.99
LMWH + antiplatelet	0	3/102 (3%)	>0.99
**Adjuvant medications**			
Hydroxychloroquine	10/18 (56%)	41/102 (40%)	0.22
Statins	9/18 (50%)	35/102 (34%)	0.20

Abbreviations: Aβ2GPI, anti‐β2‐glycoprotein‐I antibody; ACL, anticardiolipin antibody; aGAPSS, Adjusted Global Antiphospholipid Syndrome Score; aPL, antiphospholipid antibody; BMI, body mass index; CRNMB, clinically relevant non major bleeding; DOAC, direct oral anticoagulant; IQR, interquartile range; LMWH, low molecular weight heparin; SLE, systemic lupus erythematosus; SD, standard deviation; VKA, vitamin K antagonist.

The proportion of patients with hypertension (56% vs. 30%, *p* = 0.04) and history of ischaemic heart disease (IHD) (22% vs. 6%, *p* = 0.04) was higher in those with new or progressive ischaemia. Multivariate analysis showed that a longer duration of follow‐up was a significant risk for the development of further ischaemic changes (odds ratio [OR] 1.43, 95% confidence interval [CI] 1.13–1.86, *p* = 0.005). There was a trend towards significance for progressive ischaemia in those with IHD (OR 5.07, 95% CI 0.90–29.69, *p* = 0.06) and ‘triple positive’ aPL (OR 2.90, 95% CI 0.89–9.70, *p* = 0.08) (see Supporting Information). There was no difference in the proportion for progressive ischaemia according to the anticoagulant used or the intensity of VKA target.

### 3.6 Indications for imaging

To assess symptoms burden in this patient cohort, the indication for imaging was reviewed. Headache was the most common symptom reported in 35 of 120 (29%) followed by cognitive impairment in 29 of 120 (24%) patients and acute sensory deficits in 28 of 120 (23%) patients. There was no difference in the proportion of symptoms between those with and without ischaemic progression (*p* = 0.5). Note that 17% (3/18) of the patients with progressive ischaemia were asymptomatic at the time with imaging. Progression‐free survival in those with and without symptoms at MRI was not statistically different (*p* = 0.16), as shown in Figure 2B, with progressive MRI changes seen in both cohorts over time.

### 3.7 Anticoagulation intensity of vitamin K antagonists upon cerebral ischaemia

Note that 80 of 120 (67%) patients received VKA at baseline MRI scan, and 63 of 80 (79%) patients received high‐intensity anticoagulation. There was no statistically significant difference in the frequency of ischaemic progression (14% HIA vs. 24% SIA, *p* = 0.36). There was also no difference in bleeding events (CRNMB: 23% in HIA vs. 24% in SIA; major bleeding: 8% in HIA vs. 6% in SIA; *p* > 0.99) between anticoagulation intensities. Further details are provided in Supporting Information.

## 4 DISCUSSION

This study reviewed the pattern and rate of change of cerebral ischaemia over time in patients with APS. We showed features for SVD were frequent in patients requiring MRI with a predominance of WMH and lacunar infarcts. Progressive changes were associated with an increased duration of follow‐up. Additionally, a history of IHD and hypertension were associated with progressive changes in APS, which are known associated factors for cerebral SVD and stroke in the general population [22, 23]. Our findings suggest that management of conventional atherosclerotic risk factors is important, in keeping with current recommendations [24, 25]. Other previously described risk factors such as concurrent systemic lupus erythromatosus and other cardiovascular risk factors, such as hypercholesterolaemia and diabetes mellitus, were not found in this cohort. ‘Triple positivity’ aPL status was more prevalent, although not statistically significant [25, 26, 27].

In patients without APS, WMH and other features of SVD are more common and extensive in patients with cerebrovascular risk factors. They are also predictive of increased risk of cerebrovascular events [28]. SVD was common feature of APS in our cohort in contrast to historical studies [29, 30]. More recently, patterns of small and lower total volume of infarcts and WMH are more predominant APS in comparison to atrial fibrillation [31]. The recognition of SVD as a pathological process is increasingly recognised in the presence of APS. In part, the increased utility of MRI and its improved technologies may be of relevance.

Although our patient cohort was not controlled, the prevalence of cerebral ischaemia appears to be higher than expected in comparison to studies with age‐comparable cohorts without APS, albeit we recognise heterogeneity in the methodology and study populations [32, 33]. The prevalence of cerebral ischaemia was also noticeable despite the majority of this cohort receiving VKA, the current standard of care for thrombotic APS [32, 33].

Cognitive impairment is a recognised feature of APS with ‘brain fog’ commonly described by patients [34]. The relevance of SVD, in particular WMH, in APS [35] and its role in associated cognitive changes [13] have increased over the last two decades. One systematic review found that WMH were associated with cognitive dysfunction, such as impairment in memory, executive function and attention, in APS [34]. The burden of WMH in APS with cognitive dysfunction was similar to multi‐infarct vascular dementia [36]. Of the 15 patients with progressive WMH in our cohort, 93% had mildly progressive ischaemia. The clinical significance of a low amount of cerebral changes is unclear and it is the authors’ opinion that interpretation should be in conjunction with clinical features, although these are often too crude. The utility of neuropsychiatric studies may be required particularly to assess for progressive deterioration in cognitive function.

We hypothesis that aPL may be associated with increased endothelial and platelet activation and possibly vasculopathy leading to a prothrombotic tendency and infarction affecting the small vessels of the brain [37, 38, 39]. Similarly, features of endothelial dysfunction and systemic inflammation have been shown in SVD without APS [40]. This may offer some explanation to the interval changes described despite adequate anticoagulation.

We reviewed the pattern of progressive ischaemic changes over time. One‐third of patients had new infarcts that were principally lacunar and in watershed territories. Watershed infarcts typically account for 10% of ischaemic strokes without APS [41]. Other causes include carotid occlusion, microembolism from atherosclerotic plaques and haemodynamic compromise. Our study suggests that aPL antibodies may also be a cause in the absence of these other conditions in this more uncommon disease pattern.

VKA treatment intensity did not have a significant impact on interval ischaemia in our cohort. There is a paucity of data focusing on antithrombotics for cerebrovascular disease in APS. A recent retrospective study showed a high proportion of patients (45%) with APS developed interval ischaemic or haemorrhagic brain lesions over average follow‐up of 5 years [42]. Note that 38% of the cohort with lower recurrence with high‐intensity anticoagulation. Another retrospective study showed that 29% of recurrent thrombotic events in APS were strokes, occurring despite anticoagulation. Risk factors were heart valve disease, systemic hypertension, elevated IgM AB2GPI, ATE at presentation and older age [23]. A meta‐analysis showed that aPL presence was not an independent predictor for recurrent stroke, although antithrombotic therapy was not considered [43]. These data suggest that the risk of cerebral ischaemic progression in patients with APS is relatively high despite anticoagulation.

We recognised several limitations to this study. Firstly, clinical information was reviewed retrospectively and from a single centre, leading to reporting bias. In terms of assessment for ischaemic progression, 73 patients were excluded because only one MRI scan was performed; therefore, this may have resulted in selection bias, with the rate of MRI changes consistent with progressive ischaemia described in this study an overestimate of the natural history of the progression rate in treated APS. Additionally, there was a shift over time from 2D to 3D FLAIR sequence and the different magnetic field strengths used, which may have had an effect on the identification of WMH. Due to the data source being from one centre with a dependence on patient and external clinician reporting, there may be underreporting of bleeding events and risk factors for progression. Similarly, information on the quality of anticoagulation using VKA shown by time in therapeutic range (TTR) was not available. To mitigate these risks, annual reviews were performed to assess for new diagnoses, complications and disease risk factors by the study centre. TTR records were routinely reviewed as part of clinical care and alternative drugs were explored if it was felt to be inadequate (TTR < 60%). Due to small frequencies, the efficacy and efficacy of other antithrombotic could not be assessed in this cohort.

We recognise that to enhance the quality of our study, more detail is required on the development of SVD and its impact on cognitive change. Moving forward, neurocognitive testing with correlation to the burden and sites of cerebral ischaemia would elucidate our findings further. Additionally, the use of comparator groups with age‐matched healthy controls and hypertension without APS should be incorporated into further prospective studies. We await the results of the current prospective ‘Rivaroxaban for stroke patients with antiphospholipid syndrome’ study (RISAPS) that use changes in WMH burden using MRI over a 24‐month follow‐up to provide further details on SVD in APS as a disease marker and to potentially provide more therapeutic options [44].

## 5 CONCLUSION

MRI changes are common in patients with APS with a predominance of SVD. Progressive cerebral ischaemia occurred in 15% of patients despite the extensive use of VKA. Additionally, cerebrovascular risk factors were more prevalent in those with progressive changes, suggesting conventional atherosclerotic risk factor management is needed in APS.

## AUTHOR CONTRIBUTIONS

Yishi Tan and Andrew J. Doyle designed the study, collected data, analysed and interpretated the data, and wrote the manuscript. Jayant Kumar and Karen A. Breen designed the study, collected data, provided analysis of the data, and critically reviewed the manuscript including the final version. Peter Somerville, Uzma Faruqi, Anicee Danaee, Pu‐Lin Luo, and Beverley J. Hunt provided input into the study design, collected data and critically reviewed the manuscript.

## CONFLICT OF INTEREST STATEMENT

The authors declare no conflicts of interest.

## CLINICAL TRIAL REGISTRATION

The authors have confirmed clinical trial registration is not needed for this submission.

## FUNDING INFORMATION

None.

## PATIENT CONSENT STATEMENT

None.

## ETHICS STATEMENT

The authors have confirmed ethical approval statement is not needed for this submission.

## Supporting information



Supporting Information

## Data Availability

Data requests can be submitted to the authors. Request will be reviewed by the authors and institution and be made available if deemed appropriate.
