# Supplementary material for: Longitudinal assessment of cerebral infarcts and small vessel disease using magnetic resonance imaging in antiphospholipid syndrome: A single‐centre retrospective study
Source: EJHaem. 2025 Feb 6;6(1):e1065. doi: 10.1002/jha2.1065 (PMC11800375; doi:10.1002/jha2.1065)
Supplement: Supplementary file 1 — Supporting Information [file JHA2-6-e1065-s001.docx]

Supplementary Materials

**Details of Magnetic Resonance Imaging**

Changes in MRI capability changes at the study centre during the revie period. 3T MRI examinations employ a higher magnetic field strength than 1.5T, allowing for higher signal to noise ratio and potentially improving the detection of cerebral lesions. As sequences were acquired over a ten-year period from 2012 to 2022, there was a shift from two-dimensional (2D) to three-dimensional (3D) FLAIR sequence in approximately 2020. The latter sequence allows for higher spatial resolution at the expense of some contrast resolution and may have a higher sensitivity for the detection of smaller white matter T2 hyperintense lesions. Separately, a shift in blood sensitive sequence from GE to susceptibility-weighted imaging (SWI) was made in 2018.

**Supplementary Table 1: Multivariate analyses of risk factors for the development of progressive cerebral ischaemia**

| **Variables** | **OR** | **95% CI** | **p-value** |
| --- | --- | --- | --- |
| Age at first scan | 1.00 | 0.95 - 1.05 | 0.99 |
| Years between positive/last scan | 1.43 | 1.13 - 1.87 | 0.005 |
| ‘Triple positive’ aPL testing | 2.90 | 0.89 – 9.70 | 0.08 |
| Use of VKA | 1.15 | 0.32 – 4.53 | 0.84 |
| History of hypertension | 2.75 | 0.76 – 10.32 | 0.12 |
| History of IHD | 5.07 | 0.90 - 29.69 | 0.06 |
| SLE | 1.28 | 0.26 - 5.28 | 0.74 |

aPL: antiphospholipid antibodiy; CI: confidence interval; IHD: ischaemic heart disease; OR: odds ratio; SLE: systemic lupus erythematosus; VKA: vitamin K antagonists

**Supplementary Table 2: Clinical outcomes in higher intensity anticoagulation versus standard intensity anticoagulation for vitamin K antagonists**

|  | **High Intensity (n=63)** | **Standard Intensity (n=17)** | **p-value** |
| --- | --- | --- | --- |
| Mean age (years - baseline scan) | 52.8 +/- 13.3 SD | 55.0 +/-12.2 SD | 0.54 |
| BMI (kg/m^2^); median (IQR) | 31.7 (25.1-34.3) [n=19] | 31.9 (26.6-38.4) [n=10] | 0.69 |
| **Ethnicity**  Caucasian  Non-Caucasian  Not stated | 47/63 (75%)  11/63 (17.5%)  5/63 (8%) | 11/17 (65%)  2/17 (12%)  4/17 (23.5%) | 0.19 |
| **Co-Morbidities**  Diabetes  Hyperlipidaemia  Systemic hypertension  IHD  Atrial Fibrillation  SLE  Smoker or ex-smoker | 2/63 (3%)  6/63 (9.5%)  26/63 (41%)  4/63 (6%)  4/63 (6%)  10/63 (16%)  5/63 (8%) | 4/17 (23.5%)  1/17 (6%)  6/17 (35.5%)  2/17 (12%)  1/17 (6%)  3/17 (18%)  3/17 (18%) | 0.02  >0.99  0.66  0.60  >0.99  >0.99  0.08 |
| **Adjuvant Medications**  Hydroxychloroquine use  Statin use | 34/63 (54%)  27/63 (43%) | 3/17 (18%)  8/17 (47%) | 0.01  0.76 |
| **Preceding Clinical Presentation**  *Arterial thrombosis*  *Venous thromboembolism*  *Obstetric morbidity* | 50/62 (81%)  29/62 (47%)  8/62 (13%) | 12/17 (71%)  8/17 (47%)  2/17 (12%) | 0.96 |
| **aPL Testing**  Lupus Anticoagulant positive  IgG/IgM Anticardiolipin positive  *Weak positive (10-40 GPL/MPL)*  *Intermed/High (>40 GPL/MPL)*  IgG/IgM Aβ2GP1 positive  *Weak positive (10-40 GPL/MPL)*  *Intermed/High (>40 GPL/MPL)*  ‘Single positive’  ‘Double positive’  ‘Triple positive’  IgG ACL titre; median (IQR)  IgM ACL titre; median (IQR)  IgG Aβ2GP1 titre; median (IQR)  IgM Aβ2GP1 titre; median (IQR) | 57/63 (90.5%)  36/63 (57%)  19/63 (30%)  17/63 (27%)  22/60 (37%)  7/60 (12%)  15/60 (25%)  25/60 (42%)  19/60 (32%)  16/60 (27%)  33.6 (12.8-149.5)  14.9 (7.6-28)  51.1 (12.7-1564.1)  4.3 (1.5-8.0) | 15/17 (88%)  9/17 (53%)  4/17 (23.5%)  5/17 (29%)  6/17 (35%)  1/17 (6%)  2/17 (12%)  10/17 (59%)  1/17 (6%)  6/17 (35%)  139.7 (6.1-433.6)  16.7 (9.1-24.9)  1241.9 (319.7-2611.4)  4.4 (1.6-17.8) | 0.68  0.87  0.55  0.27  0.06  0.49  0.67  0.74  0.27  0.66 |

Aβ2GPI: anti β2-glycoprotein-I antibody; aCL: anticardiolipin antibody; aPL: antiphospholipid antibody; BMI: body mass index; DOAC: direct oral anticoagulant; INR: International Normalised Ratio; intermed: intermediate; LMWH: low molecular weight heparin; SD: standard deviation; IQR: interquartile range
